# A Neural Network Model With Gap Junction for Topological Detection

**DOI:** 10.3389/fncom.2020.571982

**Published:** 2020-10-14

**Authors:** Chaoming Wang, Risheng Lian, Xingsi Dong, Yuanyuan Mi, Si Wu

**Affiliations:** ^1^Peking-Tsinghua Center for Life Sciences, School of Electronics Engineering and Computer Science, IDG/McGovern Institute for Brain Research, Peking University, Academy for Advanced Interdisceplinary Studies, Beijing, China; ^2^Hefei Comprehensive National Science Center, Institute of Artificial Intelligence, Hefei, China; ^3^Chinese Institute for Brain Research, Beijing, China; ^4^Center for Neurointelligence, School of Medicine, Chongqing University, Chongqing, China

**Keywords:** global first, topological perception, gap junction, electrical synapse, subcortical pathway, ipRGCs, alpha RGCs, superior colliculus

## Abstract

Visual information processing in the brain goes from global to local. A large volume of experimental studies has suggested that among global features, the brain perceives the topological information of an image first. Here, we propose a neural network model to elucidate the underlying computational mechanism. The model consists of two parts. The first part is a neural network in which neurons are coupled through gap junctions, mimicking the neural circuit formed by alpha ganglion cells in the retina. Gap junction plays a key role in the model, which, on one hand, facilitates the synchronized firing of a neuron group covering a connected region of an image, and on the other hand, staggers the firing moments of different neuron groups covering disconnected regions of the image. These two properties endow the network with the capacity of detecting the connectivity and closure of images. The second part of the model is a read-out neuron, which reads out the topological information that has been converted into the number of synchronized firings in the retina network. Our model provides a simple yet effective mechanism for the neural system to detect the topological information of images in ultra-speed.

## 1. Introduction

It has been a long-standing debate in the field concerning whether feature analysis in visual information processing goes from local to global, or from global to local (Palmer, 1999; Chen, 2005b). The former claims that the primitives of visual processing are local features of objects. This view has successfully explained a large number of experimental phenomena (Hubel and Wiesel, 1959; Treisman and Gelade, 1980; Marr, 1982; Hubel, 1988; DiCarlo et al., 2012), but failed to account for others where visual systems show superior sensitivity to global features, e.g., the topological perception (Chen, 1982, 2005b), the configural-superiority effect (Weisstein and Harris, 1974; Navon, 1977; Pomerantz et al., 1977), the holistic processing of face and objects (Farah et al., 1998; McKone et al., 2007; Goffaux et al., 2010; Taubert et al., 2011; Bona et al., 2016), and Gestalt psychology (Wagemans et al., 2012). On the other hand, the global-to-local view states that in the visual processing, global features of objects are processed first, which subsequently guide the processing of local features (Hegdé, 2008).

**Figure 1:**
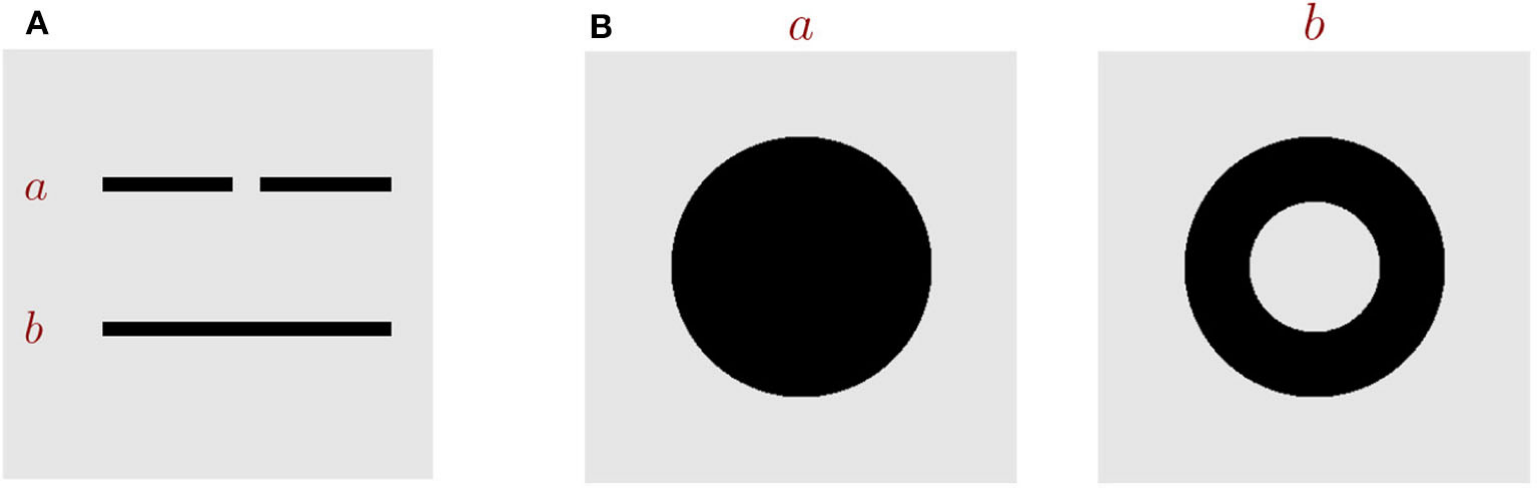
Key topological properties. **(A)** Images *a* and *b* are topologically different in the property of connectivity. **(B)** Images *a* and *b* are topologically different in the number of holes.

In the framework of global-to-local processing, Chen et al. went one step further to argue that the global nature of visual perception can be described by topological invariants and that the global precedence actually is topological primacy (see review Chen, 2005b). Topology is defined as the geometric properties which are preserved under continuous transformations, such as stretching and bending (Armstrong, 2013), and important topological properties include connectivity and the number of holes. Two shapes are called topologically different, as long as they differ in either the connectivity or the number of holes (Figure 1). Over decades, accumulating evidences on adults, infants and animals have demonstrated that visual systems are highly sensitive to topological features. The pioneering work of Chen (1982) first revealed that in the adult human visual system, the topological perception is prior to perceptions of other geometrical properties. Specifically, under 5-ms stimulus presentation, he found that subjects could discriminate a disc vs. a ring (which are topologically different) with a much higher accuracy than a disc vs. a square or a triangle (which are topologically same but different in other geometrical properties). Later, in other tasks, including multiple-object tracking (Zhou et al., 2010) and long-range apparent motion perception (Zhuo et al., 2003), Chen et al. further confirmed that the human visual perception is indeed sensitive to the connectivity or the hole of stimuli. The studies on infants also support the precedence of topological perception (Piaget and Inhelder, 1956; Darke, 1982; Chien et al., 2012; Kibbe and Leslie, 2016). It was found that newborns, even as young as few days old, display the preference of using the topological information to discriminate objects (Turati et al., 2003). Furthermore, animal studies provide more evidence to support the notion that topological perception is primitive in the visual processing. For example, Chen et al. (2003) found that honey bees with small brains have the ability to distinguish patterns that are topologically different after only a few trials learning, and they could even generalize the learned figure to novel patterns never seen before. Experiments from other researchers also demonstrated that chicks (Versace et al., 2016) and pigeons (Watanabe et al., 2019) use topological features as cues for discriminating objects.

Altogether, it suffices to say that topological properties are essential for visual perception, and very likely, they are the primitives of visual perception. Computationally, using topological features to represent and characterize objects has advantages, as it provides a relatively stable way to represent objects under transformations like stretching, rotation, or distortion. Although it is coarse, topology discrimination enables animals to detect the presence of objects rapidly without detailed local feature analysis, and this is crucial for animals to survive in natural environments.

Despite topological perception has been well-documented in the literature, the detail mechanism of how the neural system implements it remains largely unclear. It is a known fact that the conventional artificial feedforward neural network has difficulty to recognize the topology of images (McClelland et al., 1987; Minsky and Papert, 1987; Wang, 2000; Chen, 2005b). Recently, a number of experimental findings indicate that topology perception in the brain is carried out via the subcortical pathway from retina to superior colliculus (SC) and then to higher cortex. First, electrophysiological studies on retinal ganglia cells (RGCs) have revealed that there exists a type of RGCs, called alpha RGCs, which are specialized to encode the global features of stimuli (Neuenschwander and Singer, 1996; Roy et al., 2017). Specifically, they found that the presentation of a contiguous stimulus, rather than disjointed local features, produced long-range synchronization among widely separated alpha RGCs (Neuenschwander and Singer, 1996; Roy et al., 2017), and importantly, the occurrence of this kind of synchronization relies on gap junctions (also called electrical synapses) between neurons (Völgyi et al., 2013; Roy et al., 2017). Second, psychophysical and neuroimaging studies on humans have indicated that SC, rather than the primary visual cortex (V1), plays an important role in topological perception. For example, Turati et al. (2003) showed that despite of their immature visual cortex, newborns of 2–3 days old were able to detect and discriminate perceptual similarity based on the hole feature. Also, it was found that aging (Meng et al., 2019) and disruption of V1 (Du et al., 2011) significantly reduced human's ability of discriminating local geometric properties, but did not affect their topological discrimination. The neuroimaging study also showed that the neural responses in SC to hole stimuli were greater than that to no-hole stimuli under the low awareness condition (Meng et al., 2018). These findings are consistent with the electrophysiological studies on SC, which unveil that the functional role of neurons in the superficial layers of SC is to encode whether there is a new object in their receptive fields (Rizzolatti et al., 1980; Girman and Lund, 2007; Ito and Feldheim, 2018), and notably, their neuronal responses to visual stimuli are irrelevant to specific features, such as direction, orientation or shape (Marrocco and Li, 1977; White et al., 2017a,b).

Inspired by the above experimental findings, we propose a simple computational model for topological perception in the brain. Specifically, the model consists of two parts. The first part is a neural network in which neurons are connected via gap junctions, and it models the neural circuit formed by alpha RGCs in the retina (Neuenschwander and Singer, 1996; Völgyi et al., 2013; Roy et al., 2017). The second part is a read-out neuron, which suggests a way for SC and higher cortical neurons (Marrocco and Li, 1977; Rizzolatti et al., 1980; Girman and Lund, 2007; White et al., 2017a,b; Ito and Feldheim, 2018) to read out the topological information extracted by the retina network. We elucidate the computational properties of the proposed network model, and demonstrate that the model is effective and robust for detecting holes in various visual stimuli as observed in human psychophysical experiments.

## 2. Materials and Methods

**Figure 2:**
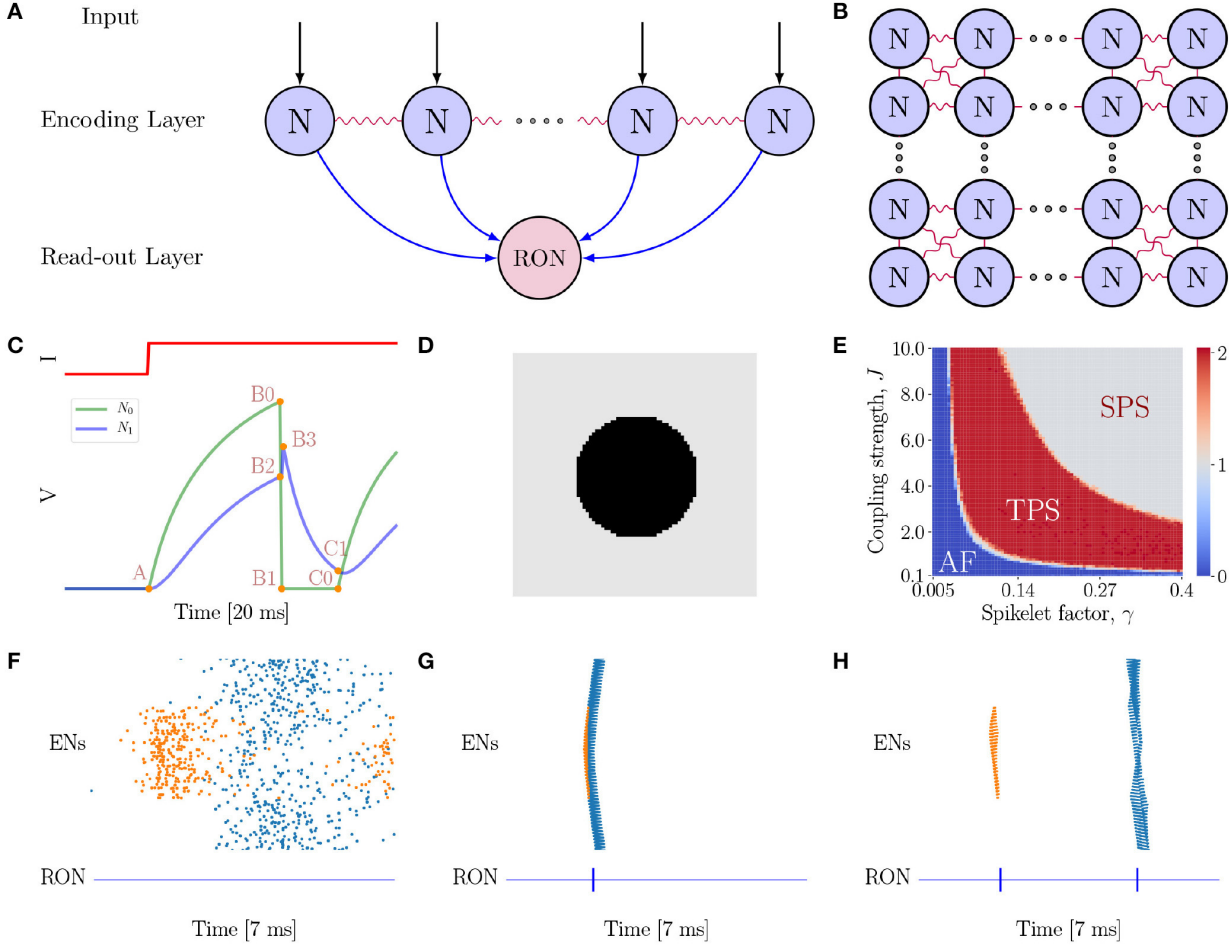
The neural network model. **(A)** The model is composed of two layers. The first layer is the encoding layer which receives external inputs, and its function is to encode the connected regions in an image. The second layer is the read-out layer, whose function is to read-out neuronal activity patterns in the encoding layer. Notably, all neurons in the first layer project excitatory synapses to the neuron in the second layer. **(B)** Neurons in the encoding layer are uniformly distributed in the space and are coupled with eight nearest neighbors with gap junctions. **(C)** Simulation of a pair of electrically coupled neurons *N*_0_ and *N*_1_. The top panel shows the external input *I* to *N*_0_, and the bottom panel presents the voltage dynamics of the neuron pair. *N*_0_ exhibits excitation and inhibition effects on *N*_1_ at different stages of the neuronal dynamics. At the A → B0 → B1 phase, *N*_0_ shows an excitatory effect to *N*_1_ (see *N*_1_ rise phase A → B2 → B3); while at B1 → C0 phase (refractory period), *N*_0_ exhibits a strong inhibitory effect to *N*_1_ (see *N*_1_ decay phase B3 → C1). **(D)** A full circle stimulus, containing two connected regions. **(E)** Parameter-space analysis of response behaviors of the network when the full circle stimulus (D) is presented. The phase plane shows three different spiking patterns which depend on the coupling strength *J* and spikelet factor γ. For each pair of (γ, *J*), the result is obtained by averaging over 10 trials. **(F)** The AF behavior of the network. *J* = 0.5 and γ = 0.05. **(G)** The SPS behavior of the network when the spikelet factor γ and the coupling strength *J* are too strong. *J* = 3.0 and γ = 0.15. **(H)** The TPS behavior of the network. *J* = 6.0 and γ = 0.25. **(F–H)** The top panel shows the raster plot of spikes in the encoding layer, while the bottom panel the spikes of RON. The abscissas and ordinates of both panels are time and neuron index, respectively. Colors indicating neurons in different groups. Specifically, coral denotes neurons on the circle, while blue denotes neurons on the background.

We consider a two-layer spiking network model (see Figures 2A,B for the network architecture illustration). The first layer is the encoding layer, which is composed of 80 × 80 encoding neurons (ENs), and the second layer is the read-out layer, which consists of only one read-out neuron (RON). RON receives excitatory projections from all ENs, and hence can read out synchronized activities in the encoding layer.

### 2.1. Neuronal Dynamics

For simplicity, all neurons in the model are implemented as leaky integrate-and-fire (LIF) models. The encoding layer receives the external inputs, and each neuron is connected to its eight neighboring neurons by electrical synapses (Figure 2B). The dynamics of an encoding neuron is given by

(1) $$\begin{array}{l} \tau \frac{dV_{i}\left(t\right)}{dt}=-V_{i}\left(t\right)+\underset{j\in N_{G}\left(i\right)}{\sum }I_{ij}^{gap}\left(t\right)+I_{i}^{ext}\left(t\right), \end{array}$$

where the subscript *i* = (1, ..., *N*) refers to the neuron index, *V*_*i*_ the membrane potential of the neuron, τ the membrane time constant, $I_{ij}^{gap}$ the current from neuron *j* transmitted through gap junction, *N*_*G*_(*i*) the set of neurons which are electrically coupled with the neuron *i*, and *I*^*ext*^ the external current from the image. Whenever *V*_*i*_(*t*) reaches a fixed threshold *V*_*th*_ (i.e., *V*_*i*_(*t*) ≥ *V*_*th*_), the neuron generates a spike and its potential is reset to the rest value *V*_*reset*_, followed by the refractory period τ^*arp*^. At the onset of the simulation, membrane potentials of all neurons are randomly initialized.

The current mediated by electrical couplings is decomposed into two parts,

(2) $$\begin{array}{l} I_{ij}^{gap}\left(t\right)=I_{ij}^{gap,sub}\left(t\right)+I_{ij}^{gap,sup}\left(t\right), \end{array}$$

where $I_{ij}^{gap,sub}$ denotes the sub-threshold current, and $I_{ij}^{gap,sup}$ the supra-threshold current, called as spikelet. The sub-threshold current mediated by electrical coupling is given by,

(3) $$\begin{array}{l} I_{ij}^{gap,sub}\left(t\right)=J\left[V_{j}\left(t\right)-V_{i}\left(t\right)\right], \end{array}$$

where *J* is the coupling strength. The supra-threshold contribution is assumed to be proportional to the gap junction strength *J* and scaled by a spikelet factor γ, which is written as,

(4) $$\begin{array}{l} I_{i}^{gap,sup}\left(t\right)=\gamma J\delta \left(t-t_{j}^{spike}\right), \end{array}$$

where $t_{j}^{spike}$ represents the spiking moment of neuron *j* and γ is a parameter controlling the contribution of a spike to the increment of neuronal potential.

The external current $I_{i}^{ext}$, which conveys the luminance level of the image, is modeled as a continuous current with a Gaussian white noise, which is written as,

(5) $$\begin{array}{l} I_{i}^{ext}\left(t\right)=\mu_{i}^{ext}+\sigma^{2}\eta_{i}\left(t\right), \end{array}$$

where μ^*ext*^ is the mean of the external input, σ^2^ the amplitude of input fluctuations, and η_*i*_(*t*) satisfies 〈η_*i*_(*t*)〉 = 0 and $\langle \eta_{i}\left(t\right)\eta_{j}\left(t^{\prime }\right)\rangle =\delta_{ij}\delta \left(t-t^{\prime }\right)$. Usually, the amplitude σ^2^ in our simulations is set to be a value, so that the noise amplitude is around 10% compared to the mean external input.

The second layer in the model is a read-out neuron (RON) (see Figure 2A), which suggests a possible way for SC neurons to read out the topological information of an image that has been extracted by the encoding layer (see more discussions in Discussion section). Specifically, we consider RON receives projections from all neurons in the encoding layer, whose dynamics is given by

(6) $$\begin{array}{l} \tau_{R}\frac{dV_{R}\left(t\right)}{dt}=-V_{R}\left(t\right)+I_{R}^{chem}\left(t\right)+I_{R}^{noise}\left(t\right), \end{array}$$

where *V*_*R*_ is the potential of RON, τ_*R*_ the time constant, $I_{R}^{chem}$ the chemical synaptic current from the encoding neurons, and $I_{R}^{noise}$ the background noise. Specifically, the current transmitted via chemical synapses is given by

(7) $$\begin{array}{l} I_{R}^{chem}\left(t\right)=\underset{j\in N_{C}}{\sum }J_{R}\delta \left(t-t_{j}-D\right), \end{array}$$

where *J*_*R*_ denotes the chemical synaptic strength, *t*_*j*_ the spiking moment of the presynaptic neuron *j*, *N*_*C*_ the set of neurons in the encoding layer, and *D* the transmission delay of chemical synapses. For simplicity, we omit the rise and decay phases of post-synaptic currents. Since the function of the read-out layer in our model is coincidence detection, we set τ_*R*_ to be sufficiently small, such that RON will fire only when a sufficient number of neuronal spikes simultaneously arrive in a short-time window. Additionally, the background noise is set to be

(8) $$\begin{array}{l} I_{R}^{noise}\left(t\right)=\mu_{R}^{noise}+\Delta \eta_{i}\left(t\right), \end{array}$$

with $\mu_{R}^{noise}$ and Δ are, respectively, the mean and the variance of the noise.

### 2.2. Simulation Experiments

In all simulations, the dynamical equations are integrated by using the Euler–Maruyama method with a fixed time-step *dt* = 0.01 ms. The network dynamics was simulated using Python, and the corresponding code the corresponding code can be available in the GitHub: https://github.com/chaoming0625/Gap_Junction_and_Topology. Parameters used in numerical simulations are reported in Table 1.

**Table 1:** Parameter of neurons, synapses, and simulation protocol.

**Parameters of the encoding neurons**	**Values**
*V*_*th*_—Spike emission threshold	10 mV
*V*_*reset*_—Reset potential	0 mV
τ—Membrane time constant	5 ms
τ^*arp*^—Absolute refractory period	3.5 ms
σ^2^—Variance of external current	1.0–2.0 mV
**Parameters of the read-out neuron**	**Values**
*V*_*th*_—Spike emission threshold	10 mV
*V*_*reset*_—Reset potential	0 mV
τ_*R*_—Membrane time constant	0.05 ms
τ^*arp*^—Absolute refractory period	0.5 ms
$\mu_{R}^{noise}$—Mean background noise	4.0 mV
Δ—Variance of background noise	0.5 mV
**Parameters of electrical couplings**	**Values**
*J*—Gap junction strength	3.0
γ—Spikelet factor	0.15
**Parameters of chemical synapses**	**Values**
*J*_*R*_—Chemical synaptic strength	0.15 mV
*D*—Chemical transmission delay	0.1 ms
**Parameters of the stimuli**	**Values**
$I_{b}^{ext}$—Value of black stimulus	20.0 mV
$I_{g}^{ext}$—Value of gray stimulus	12.0 mV

## 3. Results

### 3.1. The Neural Network Model With Gap Junction

In our proposed model (Figures 2A,B), gap junction plays a key role for topological detection. The neuronal interaction mediated by gap junction exhibits two prominent properties, as illustrated in Figure 2C. Firstly, once a neuron fires, the spike generated by it will increase the potentials of the connected neurons rapidly, and this tends to synchronize coupled neurons in the network. Secondly, after firing, the neuron falls into the refractory period with a deep low potential, which induces strong negative currents to the connected neurons, and this tends to inhibit the firing of coupled neurons [note that $I_{ij}^{gap,sub}\left(t\right)=-V_{i}\left(t\right)$, when *V*_*j*_ = 0]. As explained below, these two salient properties give rise to characteristic network responses which are differentiable with respect to connected and non-connected regions in an image.

As an example, consider a full black circle as in Figure 2D is presented to the network. The whole image consists of two connected regions, the circle and the background, which have different luminance levels. In our model, neurons covering a connected region (having the same luminance level) receive the same external input. We find that the network exhibits three response behaviors depending on the properties of gap junction (Figure 2E), which are: (1) Asynchronous Firing (AF, Figure 2F), i.e., all ENs fire independently and irregularly. This happens when both the spikelet factor γ and the coupling strength *J* are too small, and the neuronal interactions are very weak, leading to that neuronal firings are largely driven by external inputs with independent noises; (2) Single Population Spike (SPS, Figure 2G), i.e., all ENs are synchronized to generate a single population spike. This happens when the spikelet factor γ and the coupling strength *J* are both too large. In such a parameter regime, the synchronization effect of gap junction is too strong, leading to that all ENs are synchronized irrespective to the different external inputs they receive. (3) Two Population Spike (TPS, Figure 2H), i.e., ENs are synchronized but meanwhile clustered to generate two population spikes depending on the external inputs they receive. This happens when the spikelet factor γ and the coupling strength *J* have appropriate values, so that, on one hand, the synchronization effect of gap junction ensures that neurons covering the same connected region (receiving the same external input) are synchronized, and, on the other hand, the inhibitory effect of gap junction ensures that the synchronized firings of neuron groups covering different regions (having different luminance levels and hence receiving different external inputs) are well-separated in time. Computationally, this is due to that the neuron group receiving the larger external input will generate synchronized firing first; after that the neurons fall into the refractory period, and they will suppress and delay the synchronized firing of the other neuron group. To accomplish the topological detection task, we set the parameters of gap junction in the regime of TPS, such that the network can on one hand, generate synchronous firings to detect connected regions, and on the other hand, stagger synchronous firings of disconnected regions.

The synchronized responses of ENs can be easily detected by RON. Due to the small time constant, RON only responds to synchronized inputs from the encoding layer. As shown in Figures 2F–H (see the lower panels), each population spike of ENs generates a single spike of RON.

### 3.2. Topological Detection of the Network

The topology of an image has two fundamental features, connectivity and closedness (the existence and the number of holes). It is straightforward to understand that our model has the capability of detecting the connectivity of an image. In response to the inputs from a connected region, the responses of the neurons covering the connected region (they receive the similar external inputs) will become highly synchronized due to their electrical couplings (Bennett and Zukin, 2004), which provides a way to encode the connectivity of the image. This is also supported by the experimental evidence, which found that long-range synchronization occurred among widely separated alpha RGCs with electrical couplings in response to a continuous stimulus, rather than to disjointed local features (Neuenschwander and Singer, 1996; Roy et al., 2017).

**Figure 3:**
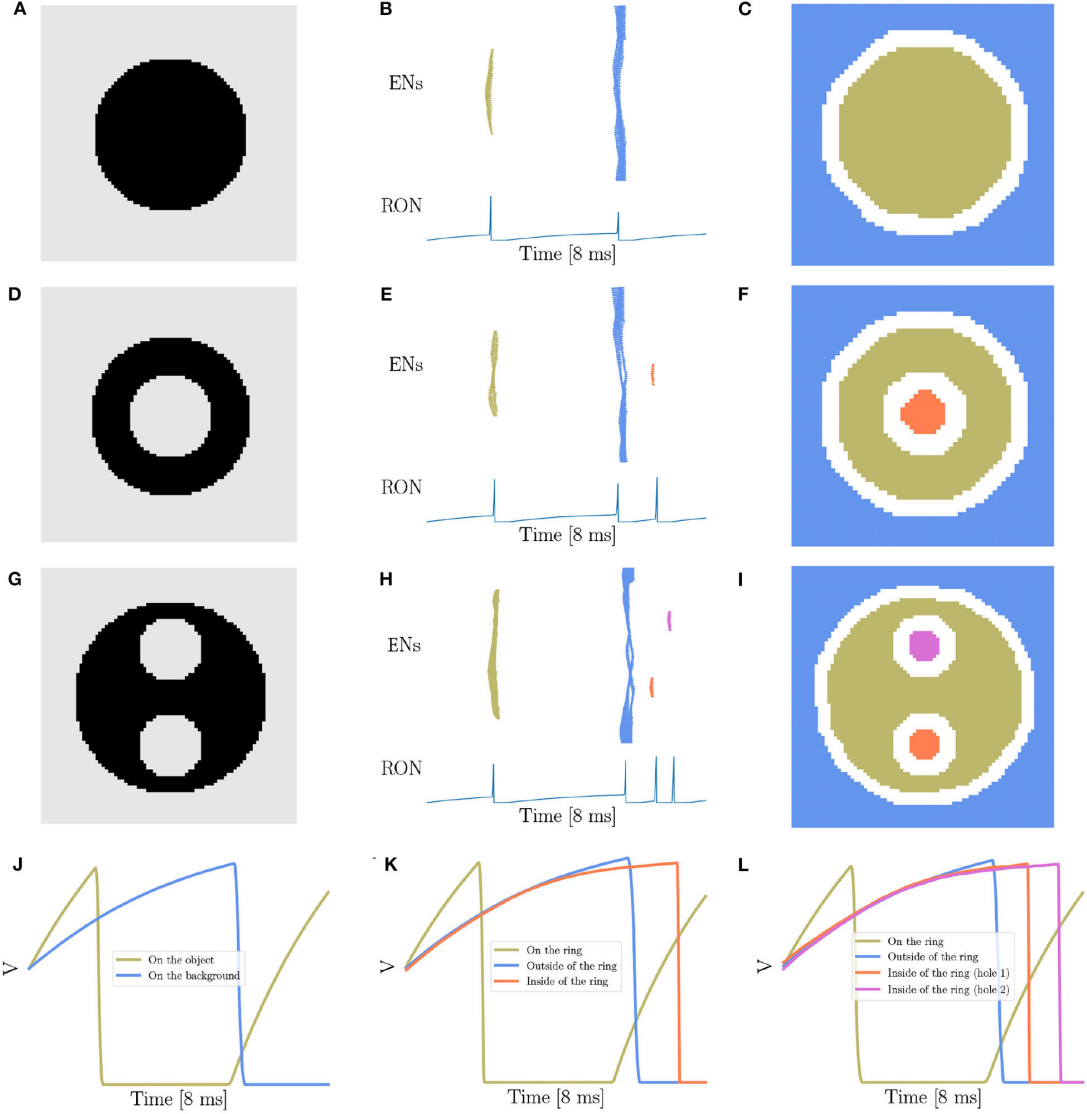
Topological detection of the network. **(A,D,G)** The images with different number of holes. **(A)** contains no hole, **(D)** one hole, and **(G)** two holes. **(B,E,H)** The evolution of network activity. **(B,E,H)** Are results when stimuli **(A,D,G)** are presented, respectively. In each subfigure, the top panel shows the raster plot of the encoding layer, and the bottom the dynamics of the membrane potential of RON. The abscissas of both panels are time, and the ordinates of the top and bottom panel are neuron index and membrane potential, respectively. **(C,F,I)** The spatial mapping of EN spikes. **(C,F,I)** corresponds to **(B,E,H)**, respectively. Neurons in the same group are shown in the same color with **(B,E,H)**. Pixels in the white color denote neurons not firing in the whole process. **(J–L)** The averaged membrane potential traces of neurons inside, on or outside of the ring when stimuli **(A,D,G)** are presented, respectively. The orange line corresponds to the neurons on the ring, the blue line the neurons on the background, and the coral and orchid lines the neurons on the holes. Parameters: *J* = 3.0 and γ = 0.15.

Therefore, the focus of the present study is to demonstrate that our network model has the capability of detecting the existence and the number of holes in an image, another key property of topology (Pomerantz et al., 2003; He, 2008; Casati, 2009; Bertamini and Casati, 2015; Zhang et al., 2019). The stimuli we used, as shown in Figures 3A,D,G, are adapted from the materials in the human and animal experiments (Chen, 1982, 2005b; Chen et al., 2003; Chien et al., 2012; Zhang et al., 2019), where Figure 3A is a solid disk without hole, Figure 3D a stimulus containing a single hole, and Figure 3G a case of two holes. Figures 3B,E,H are the corresponding network responses to the stimuli, and Figures 3C,F,I are the illustrations of synchronized neuronal responses in ENs.

Overall, we show that the number of holes in an image is encoded by the number of synchronized responses (population spikes) in the encoding layer, which are further readout by the number of spikes of RON. For example, presentation of Figure 3A produces two population spikes of ENs and two spikes of RON (Figures 3B,C), while presentation of Figure 3D produces three population spikes of ENs and three RON spikes (Figures 3E,F). Notably, although the stimulation value (the luminance level) of the hole (inside the ring in Figure 3D) is the same as that of the background (outside the ring in Figure 3D), the synchronized response of the neurons covering the hole (the orange spikes in Figures 3E,F) always lags behind that of the neurons covering the background (the blue spikes in Figures 3E,F). This property comes from that compared to the neurons outside the ring, the neurons inside the ring receive stronger inhibition from the neurons on the ring (see more detailed analysis in the below). Moreover, we observe that presentation of Figure 3G (containing two holes) reliably produces four population spikes of ENs and four RON firings (Figures 3H,I).

To reveal the underling mechanism, we look at the dynamics of neurons inside, on, and outside the ring. Results are shown in Figures 3J–L. First, we see that because of receiving a stronger stimulation than those on the background or inside the ring, the neurons on the ring (black pixels) generate the first population spike; afterwards those neurons fall into a deep and relatively long-lasting refractory period (see the voltage trace in khaki color illustrated in Figures 3J–L). Second, during the refractory period of ring neurons, while the neurons inside and outside the ring all receive inhibitions from the ring neurons, inside neurons tend to receive stronger inhibitions than outside ones (see the voltage traces in blue and orange color shown in Figure 3K). Therefore, under the condition of receiving the same level of stimulation, the neurons inside the ring always generate a population spike before the neurons outside the ring. Third, for an image containing two holes having exactly the same size and surroundings, although the neurons inside two holes receive the same external input and lateral inhibition from surrounds, they still tend to fire at different moments due to receiving independent noises (see the average voltage dynamics in orange and orchid color in Figure 3L).

Notably, because of noises, the network response varies over trials. In the case of discriminating two holes from one hole, we observed a successful rate of 70%. This probabilistic behavior is in agreement with the observation of human psychophysical experiments, which showed that the topological detection of humans is also probabilistic when images are only briefly presented in <10 ms, e.g., the successful rate of discriminating hole from circle is about 64.5% (Chen, 2005b). For visualizing the detailed spatio-temporal voltage dynamics when the stimuli (Figures 3A,D,G) are presented, please refer to Supplementary Videos 1–3. Note that, for simplicity, we have only presented the results for images with shape luminance level changes. We check that our model works equally well when the luminance intensity of the image changes smoothly (see Supplementary Figure 1).

In summary, we demonstrate that the synchronization and lateral inhibition effects mediated by gap junctions enable the network to encode the number of holes in an image into different numbers of population spikes of ENs, which provides a reliable cue for the neural system to read out the topology information of an image.

### 3.3. Topological Detection Is Invariant to Variations of Shape and Spatial Frequency

To confirm that our network model can really detect the topological property of closedness, we vary the stimulus to various forms, while keeping their topological property unchanged.

**Figure 4:**
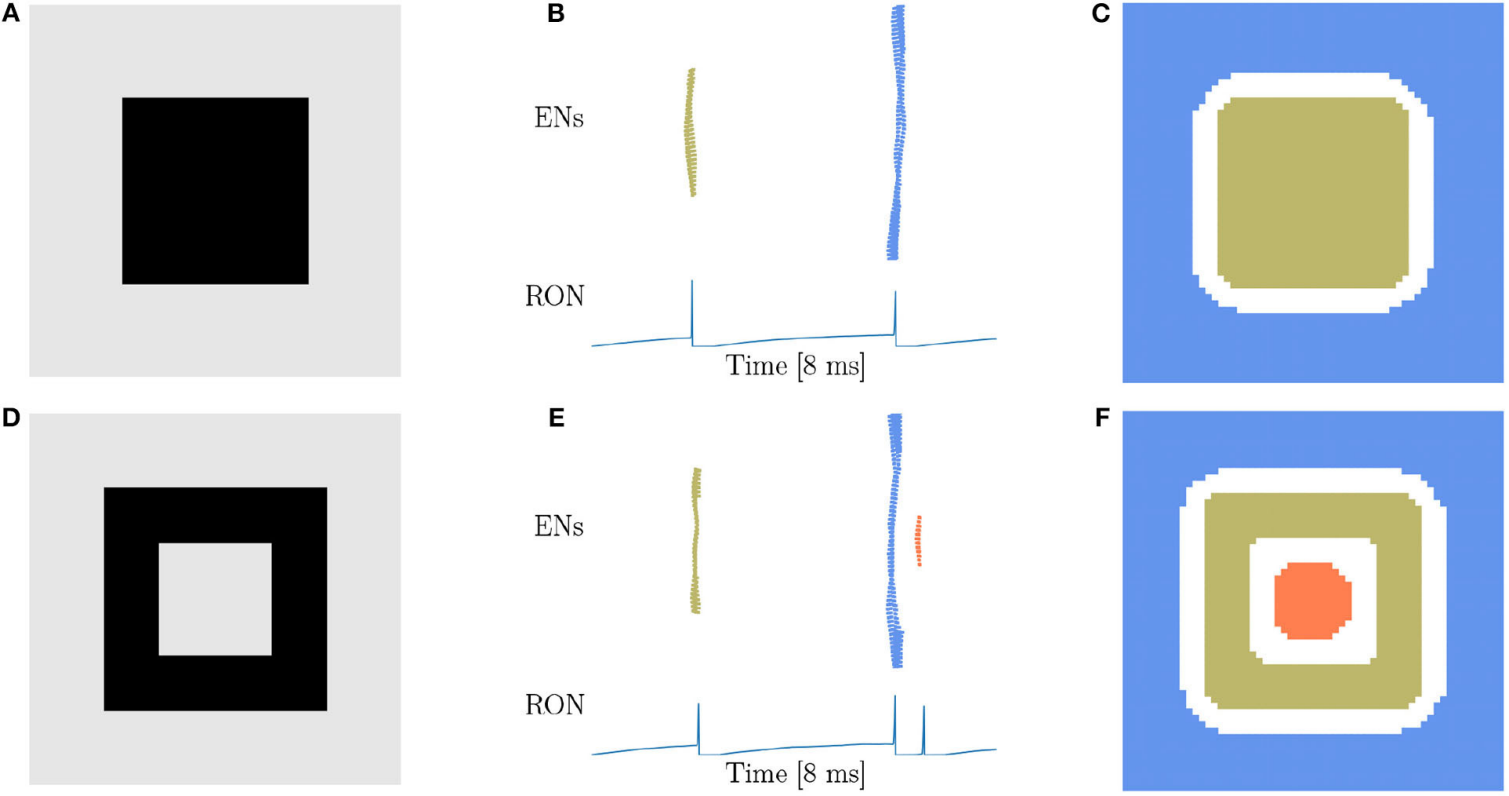
Topological detection with respect to shape variation of images. **(A,D)** Image of square shape. **(A)** A solid square. **(D)** A hollow square. **(B,E)** Population spikes of ENs (top panels) and the voltage dynamics of RON (bottom panels). **(C,F)** Spatial activities of EN neurons. Figure legends are the same as in Figure 3. Parameters: *J* = 3.0 and γ = 0.15.

From our intuitive experience, circle, square, triangle, and cross are quite different figures, but from the viewpoint of topology, they are equivalent. Therefore, the characteristic of network responses for topological detection should be the same. We first conduct experiments on a solid (Figure 4A) and a hollow squares (Figure 4D), and find that the network responses are exactly the same as when the disk (Figure 3A) and the ring (Figure 3D) are presented, that is, two population spikes of ENs and two RON spikes are generated for the stimuli without hole (comparing Figures 3B,C with Figures 4B,C), and three population spikes of ENs and three RON spikes are generated for the stimuli with one hole (comparing Figures 3E,F with Figures 4E,F). Furthermore, we perform experiments on a solid triangle (Supplementary Figure 2A), a hollow triangle (Supplementary Figure 2B), and a cross (Supplementary Figure 2C), and get the same result. Overall, these results confirm that the network response varies with the topology, rather than the shape of the stimulus.

**Figure 5:**
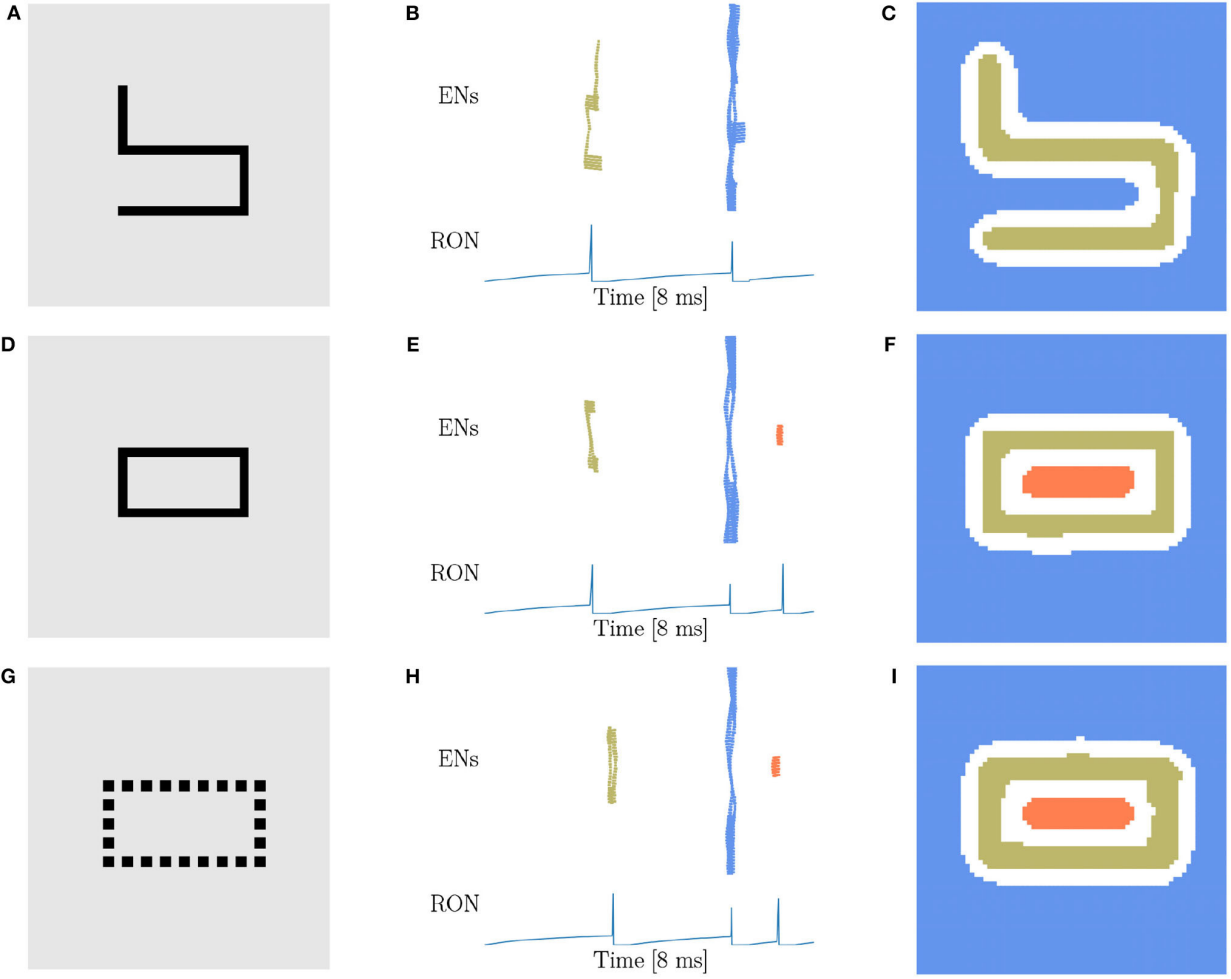
Topological detection with respect to variations of spatial frequency of images. **(A,D,G)** Images with different spatial frequencies. **(A)** An image made of four line segments without hole. **(D)** An image made of the same four line segments as in **(A)** but containing one hole. **(G)** An image shaped like **(D)** but comprised of discrete dots. **(B,E,H)** Population spikes of ENs (top panels) and the voltage dynamics of RON (bottom panels). **(C,F,I)** Spatial activity of EN neurons. Figure legends are the same as in Figure 3. Parameters: *J* = 3.0 and γ = 0.15.

Based on the finding of Carlson et al. (1984) that geometrical illusions are not primarily a consequence of low spatial frequencies and the suggestion of Chen (2005a) that low spatial frequencies are not likely to be critical to perceptual organization in general, we try to figure out whether the spatial frequency will affect the network behavior. Considering that the stimuli used above are all in low spatial frequencies (LSF), we construct new stimuli (Figures 5A,D,G) in high spatial frequencies (HSF), which are adapted from the materials used in human experiments (Carlson et al., 1984; Chen, 2005b). Figures 5A,D are made of exactly the same four line segments, while they are topologically different. We find that the network response doesn't vary with the spatial frequency. Specifically, the stimulus without hole persistently produces two population spikes of ENs and two RON spikes (Figures 5B,C), whereas the stimulus with one hole reliably generates three population spikes of ENs and three RON spikes (Figures 5E,F). We also try stimuli of triangle-shape and obtain the same result, see Supplementary Figure 3. Furthermore, we generate a stimulus composed of discrete dots (Figure 5G), which is similar to the figures in Carlson et al. (1984) and is free of low spatial frequencies. We observe that the network model displays the same response property as when the continuous line is presented (comparing Figures 5H,I with Figures 5E,F). Altogether, these results indicate that the hole detection property of our model is rather robust to the variation of spatial frequencies of images.

In above, we demonstrate that the topological detection of our network model is rather robust to the variations of shape and spatial frequency of images. It is also straightforwardly understandable that our network model is invariant with respect to the position shift, rotation, and distortion of an image, as they all generate the same number of population spikes of ENs depending only on the number of holes in the image. Thus, our network model does have the capability of detecting the topological property of an image.

### 3.4. Sensitivity of Topological Detection

**Figure 6:**
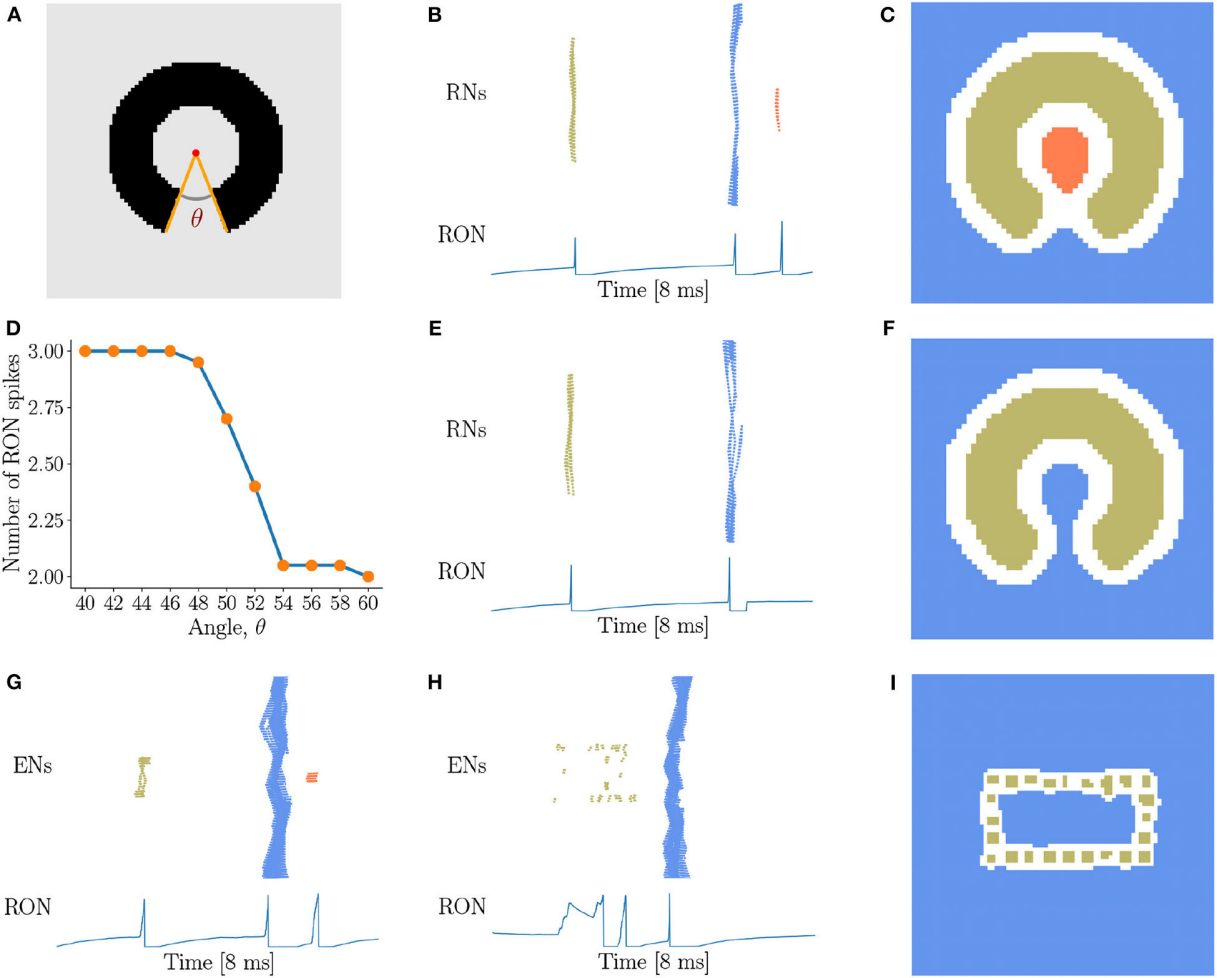
Sensitivity of topological detection. **(A)** An example of a ring with a breach, whose degree is θ. θ = 40° is shown. **(B,C)** The network activity in response to a ring with a small breach, where ENs generate three population spikes and RON produces three spikes. *J* = 3.0, γ = 0.15, θ = 40°. **(D)** The average number of RON spikes vs. the breach size. The transition occurs sharply around 50°. The results are obtained by averaging over 20 trials. **(E,F)** The network activity in response to a ring with a big breach, where ENs generate two population spikes and RON produces two pulses. *J* = 3.0, γ = 0.15, θ = 54°. **(G–I)** The response properties of the network with a varied coupling range, where each neuron is connected to its four nearest neighbors. **(G)** The image of Figure 5D is presented. **(H,I)** The image of Figure 5G is presented. Parameters: *J* = 3.0, γ = 0.20. **(B,C,E–I)** Figure legends are the same as Figure 3.

In above, we have demonstrated that our network model is able to detect the existence of holes in an image, i.e., the closure of a region. In practice, there always exists a threshold of gap below which we perceive disconnected segments as connected. Therefore, we are going to investigate how our network model is sensitive to the gap size in topological detection. We present incomplete rings with different degrees of breach (Figure 6A) to the network, and observe that with the small size of breach, the network outputs three RON spikes (Figures 6B,C). However, when the breach size θ gradually increases, the network suddenly “recognizes” that the image has no hole (see Figure 6D), i.e., ENs only generate two population spikes (Figures 6E,F). This is straightforwardly understandable, as the breach increases, the activities of the neurons inside and outside the ring become more and more synchronized due to more and more direct interactions between them, and eventually the population spikes they generate merges to a single one (see Figures 6E,F). Interestingly, we find that this transition occurs sharply, which is around the breach size of 50° at the current parameter setting (see Figure 6D). We confirm that although the value of the transition point may vary with the parameters, this sharp transition behavior always holds (see Supplementary Figure 4). This property can serve as a prediction of our model testable in human psychophysical experiments.

Furthermore, we test how the coupling range of gap junction affects the sensitivity of topological detection. We construct a network model in which each neuron is connected with its four nearest neighbors. We first confirm that the model has the capability of detecting a hole in an image, see the network response in Figure 6G when the stimulation of Figure 5D is presented. However, we also observe that when the image composed of dotted lines as shown in Figure 5G is presented, the network is unable to generate synchronous firing, but is rather in the state of irregular firing (see Figure 6H), and the network response can no longer stagger the hole and the background. This result tells us that the coupling range of gap junctions between neurons strongly affects the sensitivity of topological detection in reality.

## 4. Discussion

In the present study, we have proposed a spiking neural network with gap junction for topological detection. Our results show that gap junction-coupled neural networks are intrinsically sensitive to the topological properties, such as connectivity, closure (Figures 3–5) or semi-closure (Figure 6) of an image. A prominent computational property of gap junction is that it promotes neuron synchronization, which endows the network with the ability of detecting connected regions in an image. Another prominent computational property of gap junction is that it mediates strong lateral inhibition between connected neurons after one of them fires. Together with the fact that neurons within a closure receive much stronger inhibition than neurons outside, the network is able to stagger the moments of neuron firings within and outside a closure, and hence produces different numbers of synchronized firings corresponding to an image having or not having holes. Overall, our model provides a simple yet effective mechanism for topological detection in neural systems. Importantly, our model captures a key behavioral characteristic of object vision, i.e., the ultra-speed object detection (Thorpe et al., 1996; Kirchner and Thorpe, 2006). It has been suggested that the human visual system has the ability of getting “gist” of a scene when the stimulus is presented as briefly as 10 ms (Hegdé, 2008). In the case of topological perception, Chen (1982) demonstrated even the stimulation duration is <10 ms, adult humans are able to discriminate the global topological difference. Our proposed model provides a simple mechanistic explanation for this kind of ultra-speed topological perception: a gap junction-coupled neural network can rapidly group those distant neurons covering the same connected region and meanwhile segregate different neuron groups covering different regions, forming a stable topological visual representation in <10 ms.

### 4.1. Biological Plausibility

Our model uses electrical synapses to synchronize distant neurons corresponding to a connected region. This is consistent with the recent experimental works which found that gap junction is important for long-range synchronization among neurons over long distances (Neuenschwander and Singer, 1996; Völgyi et al., 2013; Roy et al., 2017). Particularly, Roy et al. (2017) found that electrical couplings between ON alpha RGCs and polyaxonal amacrine cells are responsible to produce the long-range correlated activity critical for global object perception. Specifically, they found that presentation of large stimuli of various shapes always produced long-range synchronization between distant ON alpha RGC pairs under electrical coupling, whereas presentation of discontinuous stimuli of several segments could not. Moreover, blockade of gap junctions diminished such kind of coherent firing. These results indicate that electrical couplings are essential for the neural representation of the image connectivity.

We propose that a retina network with electrical coupling is capable of encoding global topological features. This is in line with the functional roles of ON alpha RGC network (Schmidt et al., 2014; Allen et al., 2019). ON alpha RGCs found by Roy et al. (2017) are actually one type of ipRGCs, i.e., M4 ipRGCs (Schmidt et al., 2011, 2014). Recently, M4 ipRGCs are found essential for full contrast sensitivity in mouse visual functions (Schmidt et al., 2014). Deletion of ON alpha RGCs in mice caused severe deficits in contrast sensitivity. Meanwhile, by constructing special patterns that are distinguishable for cones but contain significant contrast for melanopsin, Allen et al. (2019) found that M4 ipRGCs in human have the capacity to encode coarse patterns and influence the appearance of everyday images. Hence, it is evident that M4 ipRGCs, which are crucial for the coarse pattern encoding and contrast sensitivity, should also be able to encode global topological patterns. However, it was reported that M4 cells have rich dendrites and exhibit non-linear spatial summation (Estevez et al., 2012). The simplified biophysics of our neurons does not capture this effect, and the functional role of dendritic computation in the M4 cells should be investigated in the future work.

If retina RGCs are able to encode global topological patterns, where and how these topological information extracted in the retina are further processed? The candidate brain area is SC. It has been long suggested that there is a type of SC neurons which is capable of global visual processing (Rizzolatti et al., 1980; Bender and Davidson, 1986). For example, Rizzolatti et al. (1980) found that some neurons in SC respond very poorly to simple visual stimuli, while produce strong and sustained discharges for all complex stimuli. In the primate, compared with the role of “feature detector” of neurons in visual cortex (like V1), this type of SCs neurons is now thought to be a class of “event detector” (Ito and Feldheim, 2018), because their responses to the visual stimuli within their receptive fields are irrelevant to the specific stimulus features, such as direction, orientation or shape (Girman and Lund, 2007; White et al., 2017a,b, 2019). One example is the recent study done by White et al. (2017a,b, 2019), in which they found that SC neurons in monkeys are capable of encoding visual saliency in a featureless manner (Marrocco and Li, 1977). Inspired by these neurobiological findings, we used a single neuron to read out each event that ENs produce coherent activity for a connected region in an image. However, our implementation of the read-out mechanism is over-simplified, because despite the existence of wide-field SC cells receiving hundreds of RGC projections (Gabbiani et al., 2001; Wang et al., 2010; Gale and Murphy, 2014), a SC neuron receiving global RGC projections is rare. Future work will consider the detailed connections between retina and SC.

### 4.2. Gap Junctions Mediate Retinal Lateral Inhibition

Lateral inhibition in the retina is thought to be crucial for visual perception (Kramer and Davenport, 2015). It has been suggested these inhibition activities are the results of retinal microcircuits which involve two inhibitory interneurons: horizontal cells (HCs) in the outer retina and amacrine cells (ACs) in the inner retina. First synaptic mechanism of lateral inhibition results from the feedback regulation mediated by HCs, which alters the neurotransmitter release in rods and cones (Wu, 1991). Later, lateral inhibition due to AC GABAergic inhibitory feedback to bipolar cells has also been observed (Feigenspan et al., 1993; Dong and Werblin, 1998; Roska et al., 2000). Furthermore, recent works suggested lateral inhibition occurs among RGCs which are indirectly mediated by spiking GABAergic wide-field ACs (Chen et al., 2016; Johnson et al., 2018). Overall, all three levels of lateral inhibition are produced by interneurons and have been shown to be closely involved in various visual processes, such as edge (contrast) enhancement (Campbell and Robson, 1968; Kramer and Davenport, 2015), spatial induction (Cook and McReynolds, 1998; Yeonan-Kim and Bertalmío, 2016), direction selectivity (Chen et al., 2016), and color processing (Schnaitmann et al., 2018). In this paper, our modeling study suggests that through gap junctions, RGCs can provide direct lateral inhibition to the coupled cells without the involvement of interneurons. This is due to that when a RGC briefly spikes, it will enter into a long refractory period, during which its connected cells via gap junctions will be strongly inhibited. This kind of lateral inhibition has been observed in Golgi cells in the cerebellar input layer (Vervaeke et al., 2010), in which a relatively deep and protracted afterhyperpolarization (one of the processes that contribute to the refractory period) in Golgi cells mediated a robust form of surround depression.

To further highlight the crucial role of gap junction-mediated lateral inhibition in topological detection, we carry out experiments by adding local GABAergic AC feedback inhibitions in the model (see Supplementary Figure 5A). Since the chemical transmission is too slow in reality, we set the synapse delay to be 0.1 ms. With such unrealistic fast feedback AC inhibition, we observe that the network behaves similarly to that without AC inhibitions (compare Supplementary Figures 5B,C with Figures 3E,F). Furthermore, to ablate the lateral inhibition of gap junctions while preserve their synchronization effect, we artificially block gap junctions when neurons are in their refractory period (setting *J* = 0). In such a way, the contribution of local chemical inhibitions is isolated. We find that: (1) when the receptive field of AC is not big enough to cover most of the hole, synchronous firings of neurons on the hole cannot be segregated from that of neurons on the background (Supplementary Figures 5D,E); (2) when the receptive field of AC is big enough to cover most of the hole, synchronous firings of neurons on the hole and the background can be well-segregated in the first 10 ms but are mixed together later on (Supplementary Figures 5F,G). Overall, our ablation study reveals that gap junction-mediated lateral inhibition is the necessary and sufficient requirement for rapid topological detection. Certainly, AC-mediated and other chemical inhibitions are also important for neural information processing, but they tend to work at different time scales and are more likely responsible for non-topological feature analysis, such as edge detection. It will be interesting to explore how different inhibitory mechanisms cooperate together to solve the coarse-to-fine feature analysis.

### 4.3. Global-to-Local Visual Processing Starts From Early Topological Detection

It is now widely agreed that visual perception takes place in a predominantly global-to-local or coarse-to-fine procedure (Bullier, 2001; Bar, 2004, 2007; Hegdé, 2008). Supporting evidence comes from the experiments using various materials, ranging from the simple stimuli [like lines, dots, gratings, and letters (Weisstein and Harris, 1974; Navon, 1977; Pomerantz et al., 1977; Watt, 1987; Hughes et al., 1996)] to complex images [such as faces (Farah et al., 1998; McKone et al., 2007; Goffaux et al., 2010; Taubert et al., 2011) and natural scenes (Parker et al., 1992, 1997; Schyns and Oliva, 1994; Lu et al., 2018)]. In this framework, the global and coarse information is processed first and subsequently activates the high-level visual cortex rather than primary visual cortex; whereafter, a feedback signal is generated and further guides the processing of the conventional local feature analysis (Bar, 2003; Bar et al., 2006). The bottom-up local feature analysis has so far been well-established, in which the visual processing begins from extracting the local features in the low visual areas followed by integrating such local features to extract more global features in the higher visual areas (Hubel and Wiesel, 1959; Treisman and Gelade, 1980; Marr, 1982; Hubel, 1988; DiCarlo et al., 2012). Later, more and more researches begin to emphasize the role of top-down facilitation in visual perception (Bar et al., 2006; Gilbert and Li, 2013). However, several questions remain elusive in this framework: how and where is such top-down facilitation ignited (Bar, 2003; Goffaux et al., 2010)? In particular, at the early visual stage, how global features are rapidly extracted?

In the case of topological perception, it has been found that the neural substrate of topological perception in humans lies in the final stage of the ventral cortical visual system, i.e., the temporal lobe (Zhuo et al., 2003; Wang et al., 2007). Moreover, on monkeys, a single-unit recording study unveiled there exists a subset of inferior temporal neurons responding selectively to hole patterns with a short latency (<100 ms) (Komatsu and Ideura, 1993). Similarly, how are such topological features extracted? What pathway does it route through to ignite the temporal lobe? Here, we hypothesize that the topological features (like “holes”) begin to be extracted in the retina. Specifically, we propose that in the retina, the alpha RGC network coupled through electrical couplings is capable of producing the topologically discriminable neural representations in a short time interval of <10 ms. We also demonstrate that such rapid and stable topological representations can be easily read-out by the SC or higher visual cortex. Our hypothesis can be partially supported by earlier two experiments (Ölveczky et al., 2003; Baccus et al., 2008). Specifically, they found that there exists a subset of RGCs specialized to distinguish local motion within the scene from the global retinal image drift due to fixational eye movements. In other words, the global motion detection begins in the retina, which supports the notion of the retinal representation of global information. In future, further detailed investigations should be carried out.

### 4.4. Related Works

The most relevant work is a pioneering model called LEGION (Wang and Terman, 1995), which was designed using the mechanisms of local excitation and global inhibition. Wang (2000) demonstrated that LEGION exhibits sensitivity to the topological connectivity, but did not investigate the detection of holes. Our model differs from LEGION in two fundamental aspects. First, the computational mechanisms are different. LEGION achieves synchronization via chemical excitatory synapses between nearby oscillators and employs a global chemical inhibitory synapse to deactivate different groups of oscillators, which are not feasible in retina; whereas, our model relies on gap functions which widely exist in the retina to synchronize and differentiate neuron groups. Second, the time courses are different. The time for LEGION to detect the topological connectivity is too slow, as the emergence of stable phase differences between objects needs multiple cycles. In contrary, our model has the ability to detect the topological property rapidly as briefly as <10 ms. Overall, our model better captures the computational nature of the retina.

## Data Availability Statement

All datasets generated for this study are included in the article/Supplementary Material. The code of the models is available on GitHub: https://github.com/chaoming0625/Gap_Junction_and_Topology.

## Author Contributions

CW, SW, and YM built the model. SW and YM supervised the project. CM, RL, and XD did the simulation and algorithm implementation. CM, SW, and RL wrote the paper. All authors contributed to the article and approved the submitted version.

## Conflict of Interest

The authors declare that this study received funding from Huawei Technology Co., Ltd. The funder was not involved in the study design, collection, analysis, interpretation of data, the writing of this article or the decision to submit it for publication.
