# Supplementary material for: A Neural Network Model With Gap Junction for Topological Detection
Source: Front Comput Neurosci. 2020 Oct 14;14:571982. doi: 10.3389/fncom.2020.571982 (PMC7591819; doi:10.3389/fncom.2020.571982)
Supplement: Supplementary file 4 [file Data_Sheet_1.pdf]

Supplementary Material

1 SUPPLEMENTARY VIDEOS

Video S1

The spatiotemporal voltage dynamics when the stimulus in Fig 3A is presented. The first frame is noisy, because neurons are initialized randomly. However, with electrical couplings, neurons' potentials rapidly become uniform. The first collective firing corresponds to the neuron group covered by the circle. Then, they enter into a refractory period. After the refractory period, the neurons covered by the background begin to fire. Notably, electrical couplings mediate temporally precise (less than 1 ms) synchrony between distant neurons covered a connected region.

Video S2

The spatiotemporal voltage dynamics when the stimulus in Fig 3D is presented. The neurons corresponding to the ring reach the firing threshold first, then go into the refractory period. In the refractory period, the neurons in the background get less inhibition than neurons in the hole. Thus, the membrane potentials of the background become higher (darker) than the hole, leading to the background produce collective firing more quickly that the hole.

Video S3

The spatiotemporal voltage dynamics when the stimulus in Fig 3G is presented. The difference between Video S2 and Video S3 lies in the firing of holes. In this video, due to the existence of noise, the neurons corresponding to two holes are independently firing.

2 SUPPLEMENTARY FIGURES

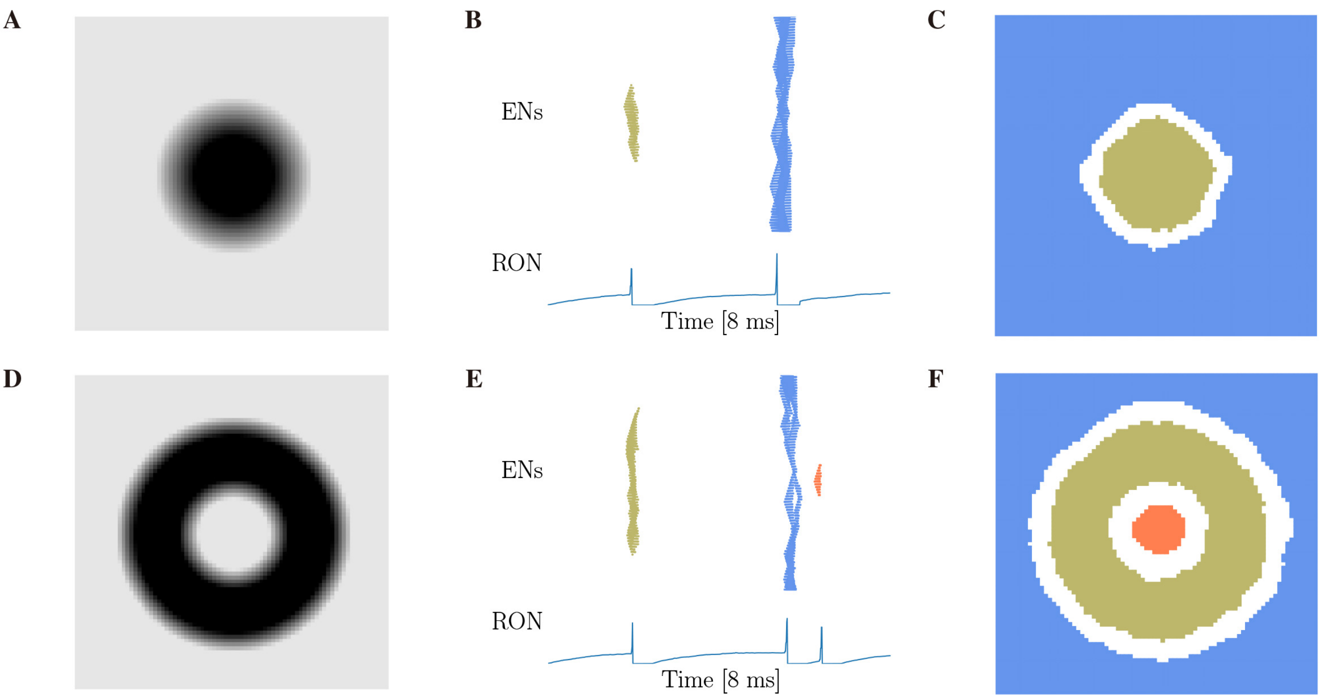

Figure S1. Topological detection of the model is robust to images with smooth luminance changes. **(A, D):** The circle and hole images with smooth intensity transition, whose topological structures are the same as that in Figure 3A and 3D. **(B, E):** The temporal evolution of neuronal dynamics. In each subfigure, the top panel shows the raster plot of the encoding layer, and the bottom panel the RON membrane potential dynamics. The abscissas of both panels are the time, and the ordinates of top and bottom panels are the EN neuron index and the RON membrane potential, respectively. **(C, F):** The spatial mapping of neuron spikes. Neurons in the same group are in the same color as in **(B, E)**. **(A-C):** The circle stimulus and the corresponding network behavior. Same as Fig 3B,C, the network produces two population of EN spikes and two RON pulses. **(D-F):** The hole stimulus and the corresponding network behavior. Same as Fig 3E,F, the network generate three population of collective EN firings and three RON spikes.

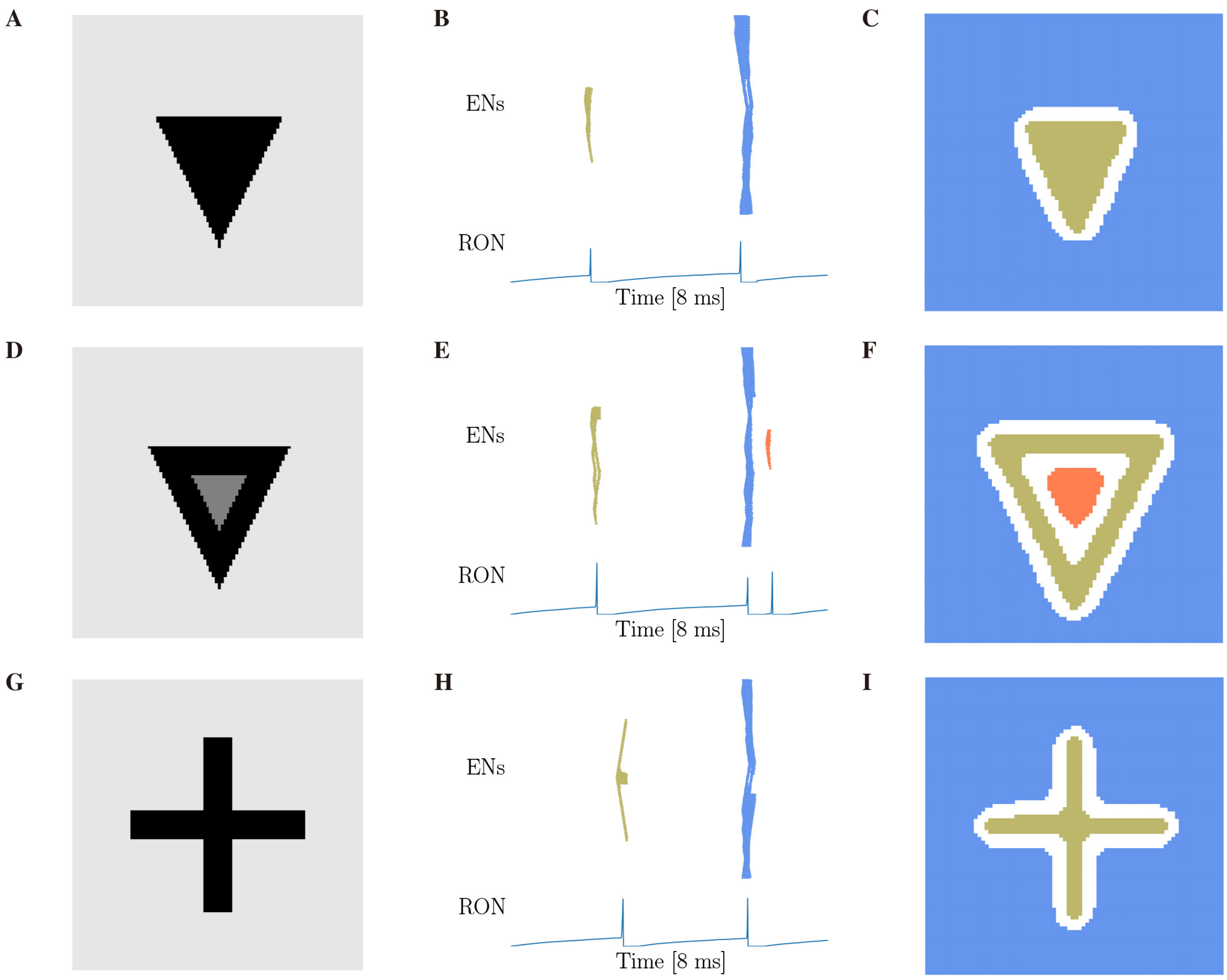

Figure S2. Topological detection is invariant to the stimulus shape. **First column (A, D, G):** The stimuli with different shapes, which respectively are the solid triangle, the hollow triangle and the cross stimulus. **Second column (B, E, H):** The temporal evolution of neuronal dynamics. **Third column (C, F, I):** The spatial mapping of neuron spikes. **First row (A-C):** The solid triangle stimulus and the corresponding network behavior. **Second row (D-F):** The hollow triangle stimulus and the corresponding network behavior. **Third row (G-I):** The cross stimulus and the corresponding network behavior.

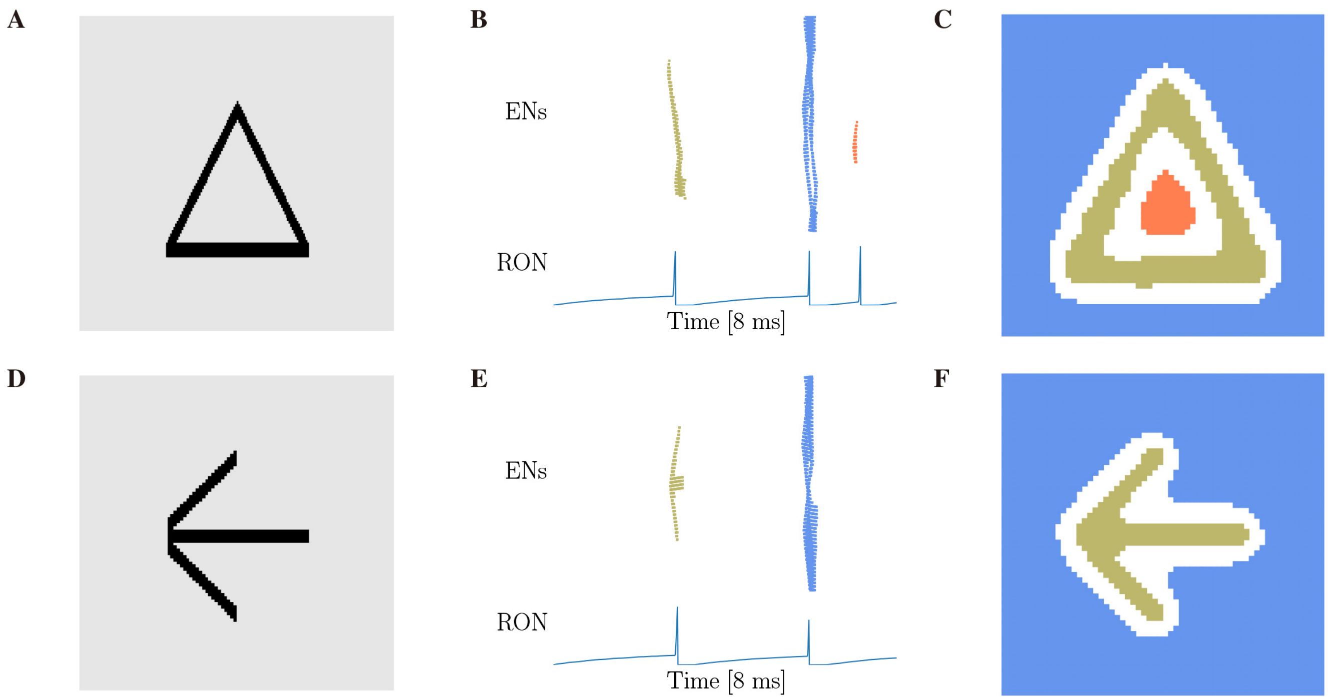

Figure S3. Topological detection is invariant to the spatial frequency. **First column (A, D):** The stimuli in high spatial frequencies (HSF). Both two stimuli are made up of three line segments, in which the former has one hole structure and the latter contains no hole structure. **Second column (B, E):** The temporal evolution of neuronal dynamics. The top panels show the raster plot of the encoding layer, and the bottom panels exhibit RON membrane potentials. **Third column (C, F):** The spatial mapping of neuron spikes. Neurons in the same group are in the same color as **second column**. **First row (A-C):** The HSF triangle stimulus and the corresponding network behavior. **Second row (D-F):** The HSF arrow stimulus and the corresponding network behavior.

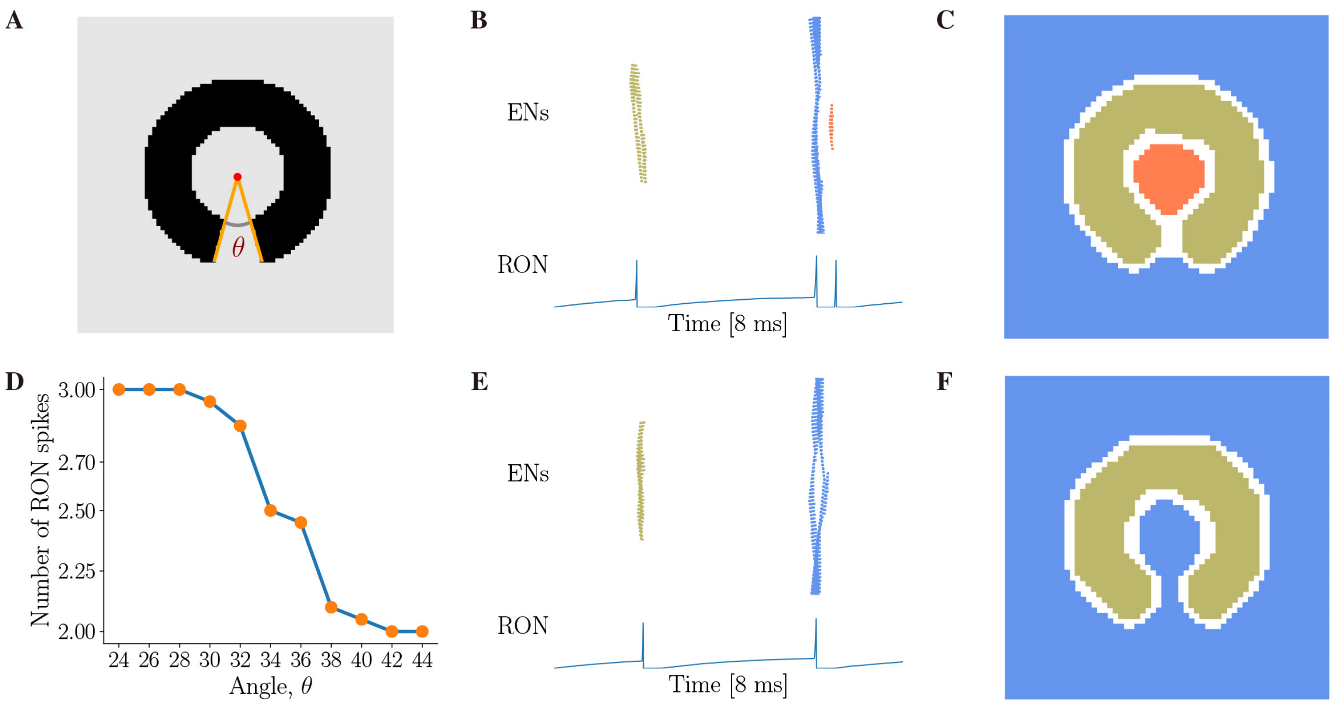

Figure S4. Hole perception for the ring with breach. **(A)** A sample image of the ring with breach, in which the angle $\theta = 30°$. **(B, C)** The behavior of the network when perceiving an incomplete ring stimulus with $\theta = 30°$, in which ENs show two synchronized population spikes and RON outputs two firings. **(D)** The curve between the breach size θ and the average number of RON spikes. The transition boundary lies between $32°$ and $34°$. **(E, F)** The behavior of the network when perceiving a image with smaller size of breach ($\theta = 40°$), in which ENs show three synchronized population spikes and RON outputs three firings. The figure legends are the same as Fig 6. Parameter settings: $J = 1.5$ and $\gamma = 0.45$.

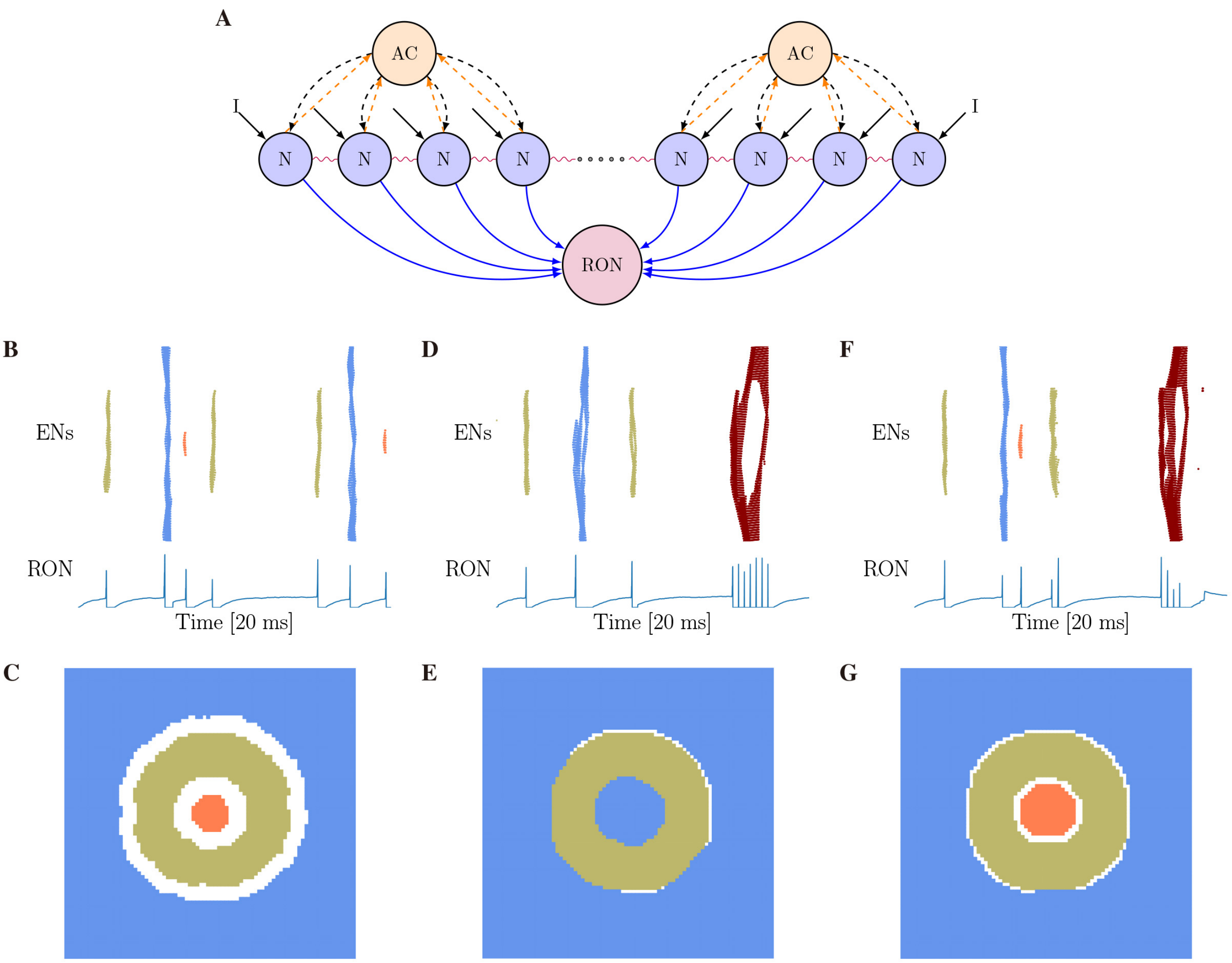

Figure S5. The network with fast feedback inhibition of Amacrine cells (ACs) and its behaviors when Fig 3D is presented. **(A)** The architecture of AC-coupled network. "N" and "RON" are RGCs and RON neurons, which are the same as Fig 2A-B. "AC" denotes Amacrine cell, which is modeled as the leaky integrate-and-fire neuron, $\tau\frac{dV}{dt} = -V + V_{rest} + I^{R2A} + \mu_i^{ext} + \sigma^2\eta_i(t)$, where $V_{rest} = 0$, membrane threshold $V_{th} = 10$, noise mean $\mu_i^{ext} = 2.0$ and noise amplitude $\sigma^2 = 0.5$. Note in first 10-20 ms, ACs do not get any inputs from bipolar cells and photo-receptors; RGCs directly receive inputs due to they are ipRGCs, and they are light-sensitive. RGCs and ACs are reciprocally connected. The dotted orange arrows are excitatory synapses of RGCs, which are given by $I_i^{R2A} = J^{R2A}\sum_k \delta(t - t_k^R - D_i^R)$. The dotted black arrows are inhibitory synapses of ACs, which are modeled as $I_i^{A2R} = J^{A2R}\sum_j \delta(t - t_j^A - D_i^A)$. In above two synapse equations, $J^{R2A} = 2.0$, $J^{A2R} = -2.0$, t_k^R and t_j^A are spike times of RGC k and AC j, D_i^R and D_i^A are synaptic delays of RGC and AC, which is set to be very fast as 0.1 ms. **(B, C)** The behavior of the network with fast AC feedback inhibition is similar to that of the network without AC inhibition (see Fig 3F). Note that in a longer time scale (20 ms), the firings of neurons on the hole and background remain separated. **(D-G)** To investigate whether the lateral inhibition of gap junction is necessary, we block gap junction in the neuronal refractory period, while keep it active in other periods. This removes the inhibition of gap junction and only keeps the inhibition of AC. **(D, E)** When the receptive field of AC is small, firings of neurons on the hole cannot be separated from those on the background, and as time goes on, all neurons' firings are mixed up (see the darkred EN spikes). In this example, 5x5 RGCs project to one AC. **(F, G)** When the receptive field of AC is big enough to cover almost the whole hole, the synchronous firing of neurons on the hole is separated from that of neurons on the background at the beginning, but later on they are still mxited. In this example, 10x10 RGCs project to one AC. Parameters: $J = 3.0, \gamma = 0.15$.
